# Current status of family physicians’ practices in geriatric medicine and associated factors in the Republic of Korea: A cross-sectional study

**DOI:** 10.1097/MD.0000000000047142

**Published:** 2026-01-09

**Authors:** Hyun-Young Shin

**Affiliations:** a Department of Family Medicine, Seoul St. Mary’s Hospital, College of Medicine, The Catholic University of Korea, Seoul, Republic of Korea.

**Keywords:** aging, family medicine, geriatrics, palliative care, primary care, rural health

## Abstract

The importance of geriatric medicine has grown significantly in recent years. This study aims to assess the current practices of family physicians in geriatric medicine in the Republic of Korea. This cross-sectional study employed online surveys targeting family medicine physicians. The questionnaire focused on the primary treatment areas of the physicians and the percentage of elderly patients in their practices. Descriptive statistical analyses were performed to interpret the data. The average age of participants was 42.6 years, with 68.2% identifying as male. Regression analysis identified several factors significantly associated with the proportion of geriatric patients, including physician age (*P* = .007), rural practice location (*P* = .001), employment in general hospitals (*P* = .012), work in geriatric hospitals (*P* < .001), involvement in hospice/palliative care (*P* < .001), and provision of pain/physiotherapy services (*P* < .001). The role of primary care in promoting healthy aging and longevity needs to be enhanced. It is crucial to cultivate primary care physicians who can lead in geriatric medicine and strengthen the essential role of primary physicians within the community healthcare system.

## 1. Introduction

As of 2024, the Republic of Korea has become a super-aged society, with over 20% of the population aged 65 and older.^[1,2]^ This demographic shift has led to a greater emphasis on geriatric medicine.^[3]^ The growing demand for geriatric specialists is clear within the medical community, prompting government initiatives focused on providing comprehensive medical care for elderly patients. These initiatives start with community-based care and incorporate chronic disease management.^[4–6]^

In 1989, the Republic of Korea established a national health insurance system, achieving a coverage rate of 74.9% by 2022.^[7]^ This system operates on a “fee-for-service” model, primarily covering medical services aimed at cost-effective disease treatment. However, it does not include provisions for disease prevention, health promotion, or quality of life enhancements. The system is structured around 3 key stakeholders: the National Health Insurance Service, which manages insurance premiums; the Health Insurance Review and Assessment, which assesses reimbursement details for medical expenses; and the medical institutions that deliver care and submit insurance claims.

Family medicine aims to provide comprehensive, continuous, and lifelong healthcare, addressing all life stages from childhood to old age.^[8,9]^ Its role is especially vital during the aging process, as it offers preventive healthcare, promotes healthy aging, and helps reduce the fragmentation of specialized care in geriatric medicine. However, several practical limitations impede the delivery of comprehensive and continuous primary care in Korea. First, specialists make up 83.2% of physicians in Korea, while only 46.4% of clinic-based physicians hold specialized certifications in primary care fields.^[10–12]^ The competition among clinics and a lack of insurance coverage that supports continuity, comprehensiveness, and family-centered care within the national health insurance system have diminished the role of primary medicine as a gatekeeper. Secondly, some primary care physicians choose to focus on areas such as health checkups, aesthetics, anti-obesity clinics, or complementary and alternative medicine (CAM).^[9]^ This trend is partly driven by concerns about physician autonomy within the current medical payment system.^[13]^

Since family medicine departments were established in Korea in 1980, there have been no studies examining the practice patterns of family physicians in providing care for geriatric patients in primary care settings. This study aims to analyze the current practices of family physicians regarding elderly patients in Korea and to identify the factors influencing these practices. The findings will help define the role of family physicians in the emerging aging society.

## 2. Methodology

### 2.1. Study population

In 2016, a comprehensive list of family medicine physicians (4058 individuals) was obtained from the databases of the Korean Academy of Family Medicine (3141 individuals) and the Korean Society of Family Medicine (1622 individuals). From these databases, members who provided their email and phone contact information were identified.

### 2.2. Questionnaire development and survey process

A self-administered questionnaire was created that included sections on respondents’ basic information, clinical practice status, workplace, medical specialty, and their reasons for choosing their area of practice. To calculate the proportion of elderly patients among the total number of patients seen by the physicians, the percentage of elderly patients reported in the questionnaire was combined with the ratios of pediatric patients (under 18 years), adults (19–64 years), and elderly patients (over 65 years). These proportions were quantified based on the average number of patients that physicians typically see in a month.

The questionnaire gathered information on several factors, including age, sex, duration of board certification in family medicine, educational degree (Bachelor’s, Master’s, or Doctoral), hospital region (rural area, small to medium city, or large city), occupation (local clinic, hospital, professor, or government), hospital level (advanced general hospital, general hospital, geriatric hospital, or clinic), working days and nights per week, monthly income levels (<5 million won, 5–10 million won, 10–15 million won, 15–20 million won, 20–30 million won, or more than 30 million won), and job satisfaction (very good, good, average, bad, or very bad). Additionally, the questionnaire assessed the physicians’ primary treatment areas, which included hospice/palliative care, aesthetic/anti-obesity clinics, pain/physiotherapy, and CAM. To finalize the questionnaire, expert meetings were conducted with family medicine professors and specialists from the Korean Academy of Family Medicine to discuss the appropriateness, specificity, and measurability of the items.

Online surveys were conducted over 4 months, from August 15 to December 15, 2016. To encourage participation, weekly emails and text messages were sent with varied dispatch times, allowing participants to respond at their convenience within a 24-hour window. The questionnaire emphasized the significance of geriatric medicine within family medicine, highlighting the value of participation in the research. This foundational study aimed to strengthen the policy capabilities of family medicine and was promoted to its members. To maintain participant anonymity, the survey questions and response time were kept concise. As an incentive, participants received a small coffee coupon for completing the survey. The study was approved by the Institutional Review Board of Myongji Hospital and included both online and offline surveys (MJH-16-097).

### 2.3. Data analysis

This cross-sectional study utilized descriptive statistical analyses to evaluate the data. We calculated the mean and standard deviation for continuous variables, and the median and interquartile range for categorical variables. Statistical significance was set at a *P*-value of <.05. To identify factors influencing the proportion of geriatric patients, we conducted a multiple linear regression analysis, incorporating variables such as age, sex, and job type (local clinic, hospital employee, professor, government, or other), region (rural area, small to medium city, or large city), type of hospital (advanced general hospital, general hospital, geriatric hospital, or clinic), working days and nights per week, income level, educational attainment (Bachelor’s, Master’s, Doctorate, or other), job satisfaction level (very good, good, average, bad, or very bad), and monthly income level. For any missing responses in the completed questionnaires, we applied the pairwise deletion method to optimize the use of available data for our analysis. All statistical analyses were performed using SAS statistical software (version 9.4, SAS Institute Inc., Cary).

## 3. Results

**Figure 1.**
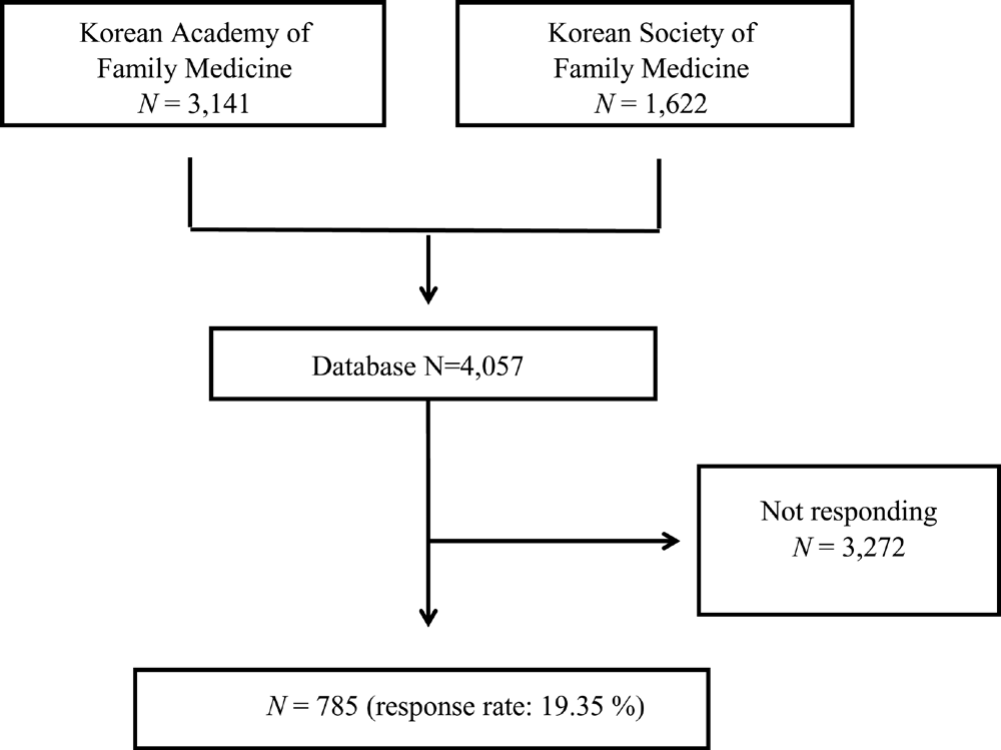
Flowchart of participant recruitment and response rate for this study.

Out of a total of 4057 family physicians, 785 potential respondents participated in the study, yielding a response rate of 19.35% (Fig. 1). The mean age of participants was 42.6 years, with males making up 68.2% of the sample. On average, participants had been board-certified for 9.9 years (Table 1). In terms of geographic distribution, 7.8% practiced in rural areas, 36.1% in small to medium cities, and 55.4% in large cities. Occupational settings included 38.7% working in local clinics, 54.0% in hospitals, 5.7% as professors, and 1.3% in government roles. Regarding hospital affiliations, 6.4% were associated with advanced general hospitals, 12.4% with general hospitals, 15.5% with geriatric hospitals, 8.8% with other hospitals, 55.5% with clinics, and 1.4% with various other types of facilities. The mean proportion of geriatric patients in the physicians’ caseloads was 50.9%.

**Table 1:** Sociodemographic and clinical characteristics of study participants.

Variables	N = 785
Age (yr)	42.6 ± 8.4
Men (number/%)	535 (68.2)
Board duration (yr)	9.9 ± 7.8
Education level (number/%)	
Bachelor	494 (63.1)
Master	203 (25.9)
Doctor	83 (10.6)
Others	3 (0.3)
Hospital region (number/%)	
Rural area	61 (7.8)
Small-middle city	283 (36.1)
Large city	434 (55.4)
Others	6 (0.8)
Job (number/%)	
Local clinic	304 (38.7)
Hospital	424 (54.0)
Professor	45 (5.7)
Government	10 (1.3)
Others	2 (0.3)
Hospital (number/%)	
Advanced general hospital	50 (6.4)
General hospital	97 (12.4)
Geriatric hospital	122 (15.5)
Hospital	69 (8.8)
Clinic	436 (55.5)
Others	11 (1.4)
Working days per week (d)	
Day	5.7 ± 0.7
Night	2.1 ± 2.3
Rate of elderly patient (%)	50.9 ± 29.2

All data are represented as mean ± standard deviation or number (%).

Table 2 displays the determinants influencing the proportion of geriatric patients in family practice, as analyzed through multiple linear regression. Key findings include: physician age (ß=0.33, *P* = .007), practice in rural areas (ß=9.87, *P* = .001), affiliations with general hospitals (ß=6.52, *P* = .012), and geriatric hospitals (ß=37.9, *P* < .001). Additionally, participation in hospice/palliative care was positively associated (ß = 13.7, *P* < .001), while involvement in aesthetic/obesity clinics (ß = −23.1, *P* < .001), pain/physiotherapy (ß = 12.7, *P* < .001), and CAM (ß = −16.6, *P* = .006) showed negative associations. Working nights per week was also negatively correlated with the proportion of geriatric patients (ß = −1.33, *P* < .001). Furthermore, an income level of 15 to 20 million won (ß = 5.92, *P* = .039) and moderate job satisfaction (ß = 3.45, *P* = .032) were identified as significant factors. The overall model accounted for 47% of the variance in the proportion of geriatric patients (*R*² = 0.47).

**Table 2:** Multiple linear regression analysis to identify variables associated with the rate of elderly patients in family practice in Korea.

	β	Standard error	*F*	*P* value
Geriatric hospital	37.9	2.8	179.7	<.001
Hospice/palliative care	13.7	3.5	15.2	<.001
Pain/physiotherapy	12.7	3.3	14.8	<.001
Rural area	9.87	3.0	10.7	.001
General hospital	6.52	2.6	6.4	.012
Income (15–20 × 10^6^ won)	5.92	2.86	4.3	.039
Satisfaction level of the job – middle	3.45	1.61	4.61	.032
Physician’s age	0.33	0.10	11.5	<.001
Working at night per week	−1.33	0.4	12.3	<.001
CAM	−16.6	6.0	7.7	.006
Esthetic, obesity clinic	−23.1	3.2	50.8	<.001

*R*^2^ = 0.47. Variables included in the linear model were age, sex, and type of job (local clinic, paid from hospital, professor, government, or others), region (rural area, small-middle, city or large city), type of hospital (advanced general hospital, general hospital, geriatric hospital, hospital or clinic), working days per week, working nights per week, income level, education level (Bachelor, Master, Doctor, or others), satisfaction level of the job (very good, good, middle, bad, and very bad), and income level per month.

CAM = complementary and alternative medicine.

## 4. Discussion

Our study highlights key findings about family medical practices for elderly patients in Korea. Factors influencing the care provided by family physicians include experience in hospice care, pain management, physiotherapy, and practice in geriatric or general hospitals. Furthermore, the physician’s age and practice location in rural areas were found to be correlated with the frequency of interactions with geriatric patients.

Historically, in the 1980s, medical students with an interest in hospice and palliative care formed a research group after obtaining their board certification in family medicine, thereby enhancing their involvement in the hospice sector.^[14]^ As the population ages and the concept of “aging in place” gains importance, family medicine can play a crucial role in delivering comprehensive medical care throughout patients’ lives. The role of family medicine in hospice care should be further expanded as society continues to age.^[15,16]^ A key public health objective during this period is to sustain community health by providing high-quality care in both rural and urban areas. However, disparities in the availability and quality of medical services between small towns and urban centers have grown. This highlights the urgent need for government intervention to ensure primary care remains accessible in vulnerable regions.^[17]^ In Japan, community-based family medicine has evolved to address the needs of an aging population, bolstered by government initiatives like scholarship programs that promote primary medical care and support regional healthcare.^[18]^ However, despite the growing number of elderly patients suffering from chronic pain, there are ongoing concerns about the ability of pain and physiotherapy specialists to meet all patient demands, given their limited numbers and the increasing costs of medications and procedures. To tackle this issue, primary care physicians should take on the management of pain-related medical issues as the first line of care, which requires thorough training in pain management during residency.^[19,20]^

Cultivating competent primary care physicians in geriatric management is crucial, as they will lead the field of geriatric medicine during the aging period.^[9,21]^ It is essential to strengthen medical care availability for the elderly and to integrate this focus into residency training and continuous medical education.^[22–24]^ Additionally, following board certification in family medicine, strategies should be developed to establish a prominent role in community-based elderly care with support from the academic community. A study by Sohn found that while family medicine residents recognized the importance of experience in geriatric medicine, their self-confidence in managing elderly patients was low.^[22]^ In response, the Korean Academy of Family Medicine has taken steps to enhance geriatric education through a core review program focused on the elderly, held twice a year, and by establishing a dedicated committee for geriatric medicine.^[25,26]^

This study has several limitations. First, it relied on a questionnaire survey that gathered self-reported data with a low response rate, potentially introducing subjective biases such as underreporting or overreporting. This low response rate may also lead to selection bias, which limits the generalizability of the findings. Second, the study is constrained by a lack of comprehensive information regarding the population samples of family medicine physicians and the standardization of participants by sex, age, and region. To address these limitations, we made efforts to minimize biases and errors in generalization when interpreting the results. Third, the questionnaire focused on outpatient treatment services rather than inpatient care. Given the strong connection between inpatient care and geriatric medicine, further research is needed to assess the current status of geriatric care among family medicine physicians who treat inpatients. Fourth, as a cross-sectional study, this research can identify associations but cannot establish causal relationships. Despite these limitations, this study is, to our knowledge, the first survey of family medicine physicians in Korea that identifies the current status of medical practices in geriatric medicine and the factors associated with it.

## 5. Conclusions

Our study identified several factors influencing the care of elderly patients by family physicians, including the physicians’ age, involvement in hospice care, pain management practices, physiotherapy, experience in geriatric or general hospitals, and work in rural areas. To promote healthy aging and longevity, it is essential to expand the role of primary medicine. This expansion will enable the delivery of cost-effective, high-quality medical services that offer comprehensive, integrated, and personalized treatment for elderly patients. In this era of increased longevity, it is crucial to invest in training primary care physicians who can effectively understand the unique medical needs of elderly patients and develop expertise in geriatric medicine management.

## Author contributions

**Data curation:** Hyun-Young Shin.

**Formal analysis:** Hyun-Young Shin.

**Investigation:** Hyun-Young Shin.

**Methodology:** Hyun-Young Shin.

**Project administration:** Hyun-Young Shin.

**Resources:** Hyun-Young Shin.

**Software:** Hyun-Young Shin.

**Supervision:** Hyun-Young Shin.

**Validation:** Hyun-Young Shin.

**Visualization:** Hyun-Young Shin.

**Writing – original draft:** Hyun-Young Shin.

**Writing – review & editing:** Hyun-Young Shin.
